# Autologous Fat Grafting Combined With Platelet-Rich Plasma in the Treatment of Female Genitourinary Aging: A Retrospective Study

**DOI:** 10.1093/asjof/ojaf165

**Published:** 2025-12-13

**Authors:** Qing-Xian Ren, Kai-Zi Li, Guo-Liang Shen, Chun-Hui Huang

## Abstract

**Background:**

Female genitourinary aging (because of childbirth, menopause, weight changes, and aging) causes genital shrinking, vaginal laxity, dyspareunia, and urinary issues, impairing quality of life. Current treatments (lubricants, hormone creams, and laser) are temporary, have drawbacks, or lack clear guidelines. Autologous fat grafting (AFG) enables safe volumization, and platelet-rich plasma (PRP) promotes tissue repair, but evidence for their combination here is scarce.

**Objectives:**

The aim of this study was to explore the clinical efficacy and safety of AFG combined with PRP in female external genitalia and vaginal rejuvenation.

**Methods:**

A retrospective study included 38 patients (January 2020 to December 2024) with genital/vaginal aging who received combined AFG (mons pubis, labia majora, G-spot, and perineum) and PRP. Postoperative evaluations were performed with the Vaginal Laxity Questionnaire (VLQ) and the Female Sexual Function Index (FSFI), and complication monitoring was conducted.

**Results:**

Postoperative VLQ scores rose from 2.45 ± 0.81 to 5.03 ± 1.15 (*P* = .027). All 6 dimensions of the FSFI (sexual interest, sexual arousal, vaginal lubrication, orgasm, sexual satisfaction, and dyspareunia) and the total FSFI score also significantly improved (all *P* < .05), with a mean total FSFI increase of 9.0 ± 2.3 points (a ≥4-point increase is clinically meaningful); 92.1% (35/38) were “very satisfied” with appearance/tightness. Only 2 (5.3%) experienced mild redness (resolved with oral antibiotics); no severe complications occurred.

**Conclusions:**

In this small cohort with limited follow-up, AFG combined with PRP treatment appears to improve the external appearance of female external genitalia and vaginal aging and enhance genital function, improve patient-reported sexual function (based on FSFI) and reduce urinary system problems.

**Level of Evidence:**

4 (Therapeutic) 
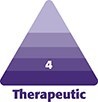

Because of factors such as childbirth, aging, weight changes, and menopause, female external genitalia (eg, mons pubis and labia majora) undergo atrophy. Additionally, conditions like vaginal relaxation, atrophic vaginitis, dyspareunia, and orgasmic dysfunction may occur, often leading to a decline in sexual life quality. Especially after menopause, women may experience a range of genital symptoms, sexual dysfunction, and urinary system issues collectively termed “genitourinary syndrome of menopause (GSM),” which significantly impacts their quality of life.^1,2^

To address these symptoms, many women seek medical products or methods to maintain their intimate health. Previous clinical studies have shown that some women use nonsurgical approaches to relieve vulvar and/or vaginal symptoms, including hormonal and nonhormonal treatments.^3^ Nonhormonal therapies, such as lubricants, moisturizers, and vitamin E, can only temporarily alleviate symptoms like vaginal dryness and itching but fail to resolve underlying pathophysiological issues and related symptoms.^2,4^ Some patients consider local hormonal therapies such as estrogen and topical testosterone; however, poor patient compliance and high recurrence rates after discontinuation are common.^2,5^ Estrogen therapy and selective estrogen receptor modulators may increase the risk of cardiovascular events such as stroke, coronary heart disease, and venous thromboembolism.^6^ Furthermore, for patients with hormone receptor-positive breast cancer, hormonal therapy may raise the risk of cancer recurrence, which is a matter of concern.^7^ New treatment methods, including laser technology, can serve as alternatives. Pulsed carbon dioxide laser therapy and monopolar radiofrequency therapy have been proven to promote neocollagenesis and angiogenesis, but these therapies require multiple sessions and lack standardized treatment protocols, necessitating further research to confirm their feasibility before routine application.^2,8-10^ Healthcare providers should actively engage in the early diagnosis and treatment of GSM symptoms, implementing personalized interventions for patients.

Autologous fat grafting (AFG), as an innovative surgical method with easy accessibility and abundant adipose tissue, has been widely used and highly evaluated in the field of plastic surgery in recent years.^11^ AFG was first proposed by Gustav Neuber in 1893, and Coleman elaborated on related techniques in the 1990s. In recent years, the scientific basis for the regenerative effects of fat grafting has been continuously deepened.^12,13^ Autologous fat can replace fillers because of its properties, such as biocompatibility, easy accessibility, low cost, abundance, and collectability. Generally, the operation is simple with few complications.^14,15^

Combined platelet-rich plasma (PRP) has emerged as a valuable adjunct in the field of plastic surgery, revolutionizing approaches to tissue regeneration and aesthetic enhancement.^16^ When utilized in combination with various plastic surgical procedures, it amplifies the regenerative potential, promoting faster tissue repair, reducing downtime, and enhancing overall outcomes.^17^ One of the key advantages of combined PRP is its autologous nature, minimizing the risk of allergic reactions or immune rejection. As research continues to explore its full potential, combined PRP is poised to remain a cornerstone in advancing the field of plastic surgery, offering safer, more effective, and natural-looking results for patients.

In this study, we introduced an AFG technique combined with PRP, which was applied in the vulvoperineal area and submucosal vaginal tissue, aiming to restore tissue volume, promote tissue regeneration in patients with intimate aging (eg, atrophy of the mons pubis and labia majora, vaginal relaxation), improve genital function, and alleviate sexual dysfunction and urinary system problems.

## METHODS

### General Information

This study was initially designed as a prospective cohort study (patients had signed the informed consent form before surgery), but because the collection of 6-month follow-up data relying on retrospective medical record review, it ultimately adopted a retrospective analysis framework. This is hereby noted.

Sample size estimation was based on the effect size (Cohen's *d* = 0.8) of the Vaginal Laxity Questionnaire (VLQ) score from previous monotherapy AFG studies.^18^ A priori power analysis using G*Power 3.1 indicated that a minimum of 32 participants was required to achieve statistical significance with *α* = .05 and *β* = .20. The present study enrolled 38 eligible participants, yielding an achieved power exceeding 85%. The informed consent signed by the patient includes clauses on “use of research data” and “publication of clinical images,” and the images have been anonymized (removing identifiable information such as faces and tattoos). All patients confirmed that there is no risk of privacy breach. The study was approved by the hospital ethics committee (no. 251243).

The main indications for including patients in this study were: (1) subjective vaginal laxity (VLQ score ≤3 points); (2) Female Sexual Function Index (FSFI) total score ≤26 points (indicating sexual dysfunction); (3) mons pubis/labia majora atrophy (physician score ≤2 points, according to Vaginal Health Index).

Inclusion criteria: (1) complete medical records, (2) aged 18 to 64 years, (3) good physical health and with requirements for genital rejuvenation, (4) meeting treatment conditions, (5) preoperative capacity to give informed consent (assessed by 2 physicians for normal cognitive function), confirmed that complete informed consent documents were retained in the medical records during retrospective analysis, and (6) informed of the study content and consenting to participate.

Exclusion criteria: (1) complicated with severe diseases of major organs, (2) cancer patients, (3) patients with hematological diseases, (4) patients with coagulation dysfunction, (5) patients with immune system diseases, (6) patients with mental illnesses, (7) pregnant or lactating women, and (8) patients with scar diathesis.

### Preparation of Autologous Fat Particles

A tumescent solution (30 mL of 2% lidocaine + 1 mL of 0.1% epinephrine + 1000 mL of normal saline + 10 mL of 8.4% sodium bicarbonate) was injected into the fat donor area (usually the thighs or abdomen) for local tumescent anesthesia. Approximately 100 mL of fat was aspirated using a 20 mL syringe connected with cannula (18-22 Fr) under negative pressure, with the aspiration area distributed in a fan shape. Rough movements were avoided to prevent damage to fat particles. After the fat extraction, apply elastic bandages to compress and dress the liposuction area. After standing, oil droplets, fibrous impurities, etc, were removed from the fat fluid. The obtained adipose tissue was filtered by centrifugation (3000 rpm) to obtain ∼50 to 100 mL of pure fat particles.

### Preparation of Platelet-Rich Plasma

According to the required amount of fat for grafting, 10 to 20 mL of the patient's peripheral venous blood was collected, and PRP was prepared using a double centrifugation method. Peripheral blood was drawn into a sterile test tube containing sodium citrate anticoagulant for the first centrifugation. After 10 min of centrifugation, the blood was divided into 3 layers: the upper and middle layers were platelet-poor plasma, highly concentrated platelets, and the lower layer was red blood cells. All supernatant was aspirated, and after removing red blood cells, the second centrifugation was performed. After 10 min of centrifugation, the liquid was again divided into 3 layers: platelet-poor plasma, PRP, and red blood cells. The supernatant and red blood cells were discarded, and a small amount of thrombin was added to activate the platelets to obtain PRP. After preparation, it was tested using an automatic blood cell analyzer (model: Sysmex XN-1000), with a platelet concentration of (1.2-1.8) × 10^6^/μL (3-5 times the platelet concentration of peripheral blood); the activator used was a 10% calcium chloride solution (concentration 0.2 mol/L), with an activation time of 5 min. PRP and autologous fat particles were mixed uniformly at a ratio of ∼1:10 and transferred to a 1 mL syringe for use.^19^

### Fat Particle Grafting

#### Processing

A stainless steel mesh sieve filtration method was used to remove part of the water, cord-like tissue was cut into pieces, and PRP and an appropriate amount of glucocorticoid were added. The corticosteroid added to the mixed solution is dexamethasone (concentration 5 mg/mL), with a dose of 0.1 mg/mL. According to the study by Serra-Mestre et al, it can reduce early postoperative inflammatory response without affecting fat survival rate.^20^

#### Injection

Injection was performed using a 17G blunt needle in layers, through multiple tunnels, and under low pressure, with a single-point injection volume ≤0.2 mL.

#### Grafting Strategies for Each Site

Mons pubis: injected into the superficial subcutaneous layer, generally 15 to 30 mL; labia majora: injected into the subcutaneous fat pad, generally 10 to 15 mL per side; G-spot: 1 to 2 mm under the mucosa, generally 2 to 4 mL; perineum: injected into 3 specific, anatomically defined potential spaces (submucosal, para-vaginal, and perineal body spaces) within the urogenital triangle, generally 20 to 30 mL. These spaces are chosen for their ability to enhance vaginal fullness, correct laxity, or improve contour, whereas the 20 to 30 mL total volume is distributed across them to balance efficacy and safety. Success depends on avoiding adjacent critical structures (urethra, rectum, blood vessels, and nerves) and aligning injection depth/volume with each space's anatomical capacity.

### Outcome Measures

Before grafting treatment and at the 6-month follow-up after surgery, the VLQ and FSFI were used to evaluate the efficacy of vaginal relaxation, self-perceived vaginal relaxation status, vaginal health level, and improvement in sexual function. The occurrence of complications was also recorded. This survey was completed by professional nurses upon the patient's admission to the hospital. The 6-month telephone follow-up after discharge was also carried out by professional nurse.

#### Vaginal Laxity Questionnaire

This is a patient self-assessment questionnaire. Patients rated their self-perceived vaginal laxity from “very loose” to “very tight” on a scale of 1 to 7 points, with lower scores indicating more severe vaginal relaxation.

#### Female Sexual Function Index

The scale consists of 6 dimensions: sexual interest, sexual arousal, vaginal lubrication, orgasm, sexual satisfaction, and dyspareunia. Each dimension has a total score of 10 points, and the sum of scores from each dimension is the total score of the scale. Higher scores indicate better sexual function experience.

### Statistical Analysis

Data are presented as the mean ± standard deviation. The VLQ and FSFI scores for each dimension and the total score were compared preoperatively and postoperatively using a paired *t* test, as the data conformed to a normal distribution (Shapiro–Wilk test, *P* > .05), so parametric tests were used. All statistical analyses were performed using GraphPad Prism software. A *P*-value of <.05 was deemed to indicate statistical significance.

## RESULTS

### Patient Satisfaction and Complications

A total of 38 patients, aged 35 to 58 years with an average of 40.8 years, were included in this study. Patient demographic and clinical characteristics are listed in Table 1.

**Table 1.** Patient Demographic and Clinical Characteristics

Characteristic	Range	Mean ± SD	Category/subgroup	*n*	Percentage (%)
Age (years)	35-58	40.8 ± 5.2	35-44 years	22	57.9
			45-54 years	13	34.2
			≥55 years	3	7.9
BMI (kg/m^2^)	19.3-28.7	23.6 ± 2.1	Normal weight (18.5-24.9)	25	65.8
			Overweight (25.0-29.9)	13	34.2
			Obesity (≥30.0)	0	0
Parity status	—	—	Nulliparous (0 deliveries)	4	10.5
			Primiparous (1 delivery)	11	28.9
			Multiparous (≥2 deliveries)	23	60.5
			Vaginal delivery (among multiparous)	18	78.3 (of 23)
			Cesarean section (among multiparous)	5	21.7 (of 23)
Smoking status	—	—	Never smoked	31	81.6
			Former smoker (quit ≥1 year)	5	13.2
			Current smoker	2	5.2
Medical comorbidities	—	—	No comorbidities	29	76.3
			Hypertension (medication controlled)	5	13.2
			Type 2 diabetes (oral meds controlled)	2	5.2
			Hypothyroidism (levothyroxine controlled)	2	5.2
HRT use	—	—	Premenopausal (no HRT use)	22	57.9
			Postmenopausal	16	42.1
			HRT user (<2 years, estrogen-only/estrogen-progestin)	7	43.8 (of 16)
			Never used HRT (postmenopausal)	9	56.2 (of 16)
Menopausal status	—	—	Premenopausal (regular cycles)	22	57.9
			Perimenopausal (irregular cycles, no amenorrhea ≥12 months)	7	18.4
			Postmenopausal (amenorrhea ≥12 months)	9	23.7

HRT, hormone replacement therapy.

All 38 patients were satisfied with the appearance of the external genitalia and vaginal tightening after surgery, among whom 35/38 (92.1%) were very satisfied. Of the total patients, 3/38 (7.9%) patients reported insufficient vaginal tightness and subsequently underwent a second surgery. During this follow-up procedure, only a repeat injection was administered to the vaginal area (dosage: 15-20 mL), with no re-treatment of the mons pubis or labia majora. They were very satisfied after the second grafting surgery. There were 2/38 (5.3%) cases of complications after surgery, both of which were local redness and swelling in the surgical area, and the symptoms disappeared after anti-inflammatory treatment with antibiotics (oral cefuroxime, 250 mg, bid for 3 days). During the 6-month telephone follow-up after discharge carried out by nurse, all patients reported good tactile sensation of the grafted area, no hard nodules, and good maintenance of fullness.

### Vaginal Laxity of Patients Before and After Grafting Treatment

After grafting treatment, the VLQ score of patients was significantly higher than that before treatment, and the difference was statistically significant (*P* = .027), as shown in Table 2.

**Table 2.** Comparison of VLQ Scores Before and After Treatment

Time	*n*	VLQ score
Before treatment	38	2.45 ± 0.81
After treatment	38	5.03 ± 1.15
*t* –value		8.625
*P* value		.027

Data were presented as mean ± standard deviation (*x* ± *s*). VLQ, Vaginal Laxity Questionnaire.

### Comparison of Female Sexual Function Index Scores Before and After Grafting Treatment

**Figure 1.**
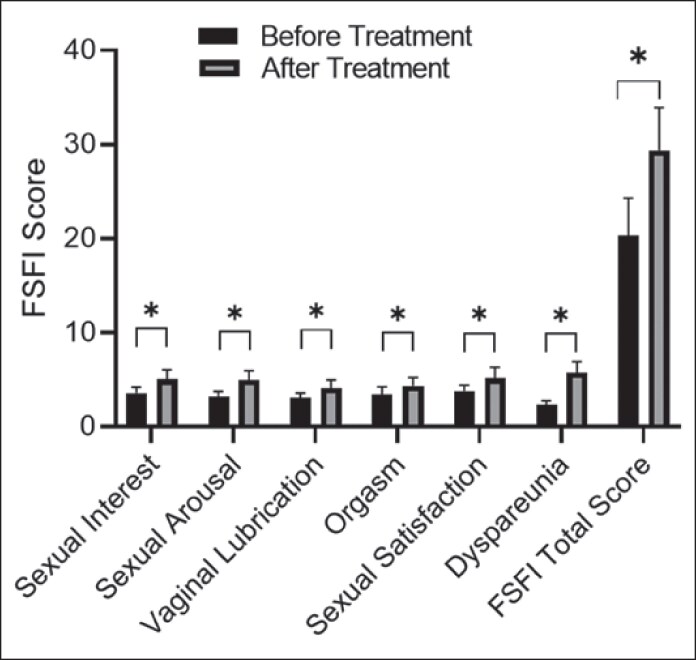
Comparison of Female Sexual Function Index (FSFI) scores of each dimension and total score before and after treatment. Columns represent mean FSFI scores of each dimension and total score for 38 patients; error bars = standard deviation (SD). “*” indicates significant difference between pre- and posttreatment scores (*P* < .05, paired *t* test).

After grafting treatment, the scores of all dimensions (sexual interest *t* = 8.23, *P* = .048; sexual arousal *t* = 10.13, *P* = .018, vaginal lubrication *t* = 6.39, *P* = .024, orgasm *t* = 7.61, *P* = .039, sexual satisfaction *t* = 15.13, *P* = .042, dyspareunia *t* = 6.17, *P* = .002), and the total score of FSFI were significantly higher than those before grafting treatment, and the differences were statistically significant (*P* = .041), as shown in Figure 1, detailed data in Table 3. According to literature reports, an increase of ≥4 points in the total FSFI score has clinical significance.^21^ In this study, the average increase in the total FSFI score postoperatively was 9.0 ± 2.3 points.

**Table 3.** Data of Comparison of FSFI Scores of Each Dimension and Total Score Before and After Treatment

Time	*n*	Sexual interest	Sexual arousal	Vaginal lubrication	Orgasm	Sexual satisfaction	Dyspareunia	FSFI total score
Before treatment	38	3.59 ± 0.61	3.17 ± 0.58	3.09 ± 0.48	3.41 ± 0.83	3.83 ± 0.59	2.27 ± 0.51	20.36 ± 3.94
After treatment	38	5.04 ± 1.02	4.97 ± 0.610	4.13 ± 0.85	4.35 ± 0.91	5.14 ± 1.21	5.75 ± 1.16	29.39 ± 4.52
*t* value		8.23	10.13	6.39	7.61	15.13	6.13	10.57
*P* value		.045	.018	.024	.039	.042	.002	.041

Data were presented as mean ± standard deviation (*x* ± *s*). FSFI, Female Sexual Function Index.

**Figure 2.**
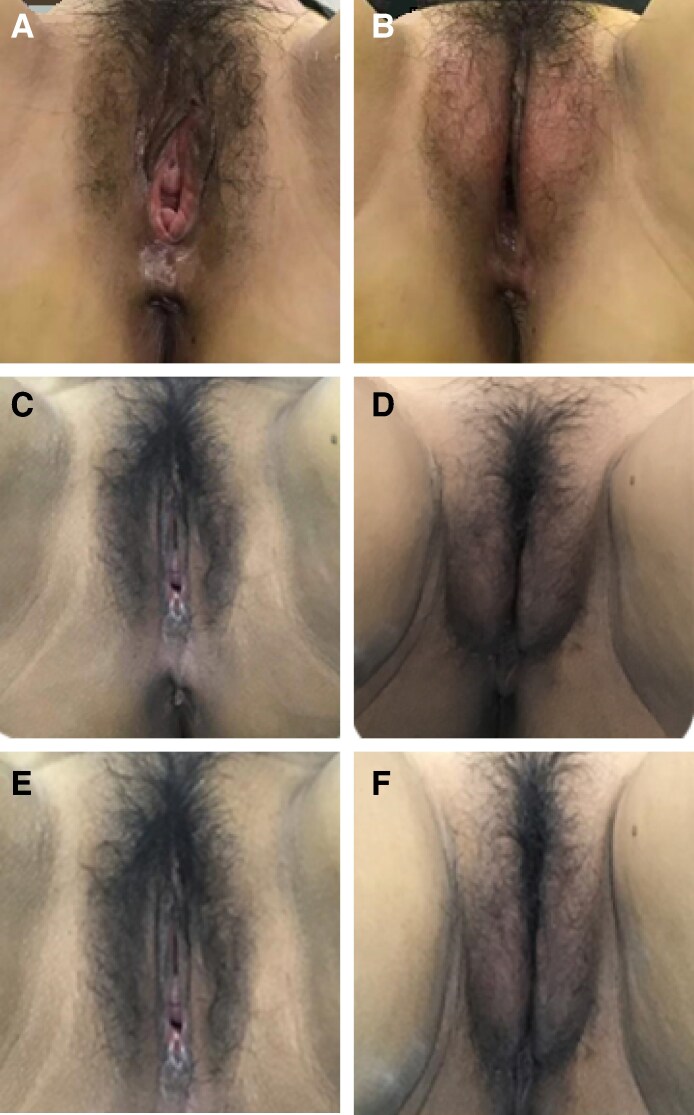
A set of representative images of following treatment with AFG and PRP combination treatment. (A) Presurgery and (B) immediate postsurgery views of a 53-year-old female. Injection details are as follows: (1) Mons pubis: injected into the superficial subcutaneous layer, with a volume of 20 mL; (2) Labia majora: injected into the subcutaneous fat pad, with 15 mL per side; (3) G-spot: injected 2 mm beneath the mucosa, with a volume of 4 mL; (4) Perineum: injected into 3 specific spaces within the urogenital triangle (submucosal, para-vaginal, and perineal body spaces), with a total volume of 30 mL. (C) Presurgery and (D) 3-month postsurgery views of a 51-year-old female. Injection details are as follows: (1) Mons pubis: injected into the superficial subcutaneous layer, with a volume of 25 mL; (2) Labia majora: injected into the subcutaneous fat pad, with 12 mL per side; (3) G-spot: injected 1 mm beneath the mucosa, with a volume of 3 mL; (4) Perineum: injected into 3 specific spaces within the urogenital triangle (submucosal, para-vaginal, and perineal body spaces), with a total volume of 25 mL. (E) Presurgery and (F) 6-month postsurgery views of the same 51-year-old female shown in C and D.

Among the cases analyzed, a representative outcome following treatment with the AFG and PRP combination—reflecting the consistent therapeutic trends documented in the study—is shown in Figure 2 for illustrative purposes.

## DISCUSSION

Our present observation revealed that AFG combined with PRP effectively addresses female external genitalia and vaginal aging by restoring tissue volume, improving genital function, enhancing sexual function, and reducing urinary system-related issues, with high safety and patient satisfaction obtained from patients’ feedback.

AFG is a commonly used technique in plastic and reconstructive surgery, involving the transfer of autologous adipose tissue from 1 part of the body to another. Coleman's technique has improved the survival rate of fat grafting, making its application more reliable and predictable. Initially, fat fillers were mainly used to correct volume deficiency and for cosmetic purposes.^22^ However, as clinicians gradually recognized that adipose tissue is a connective tissue containing mesenchymal stem cells, which can divide indefinitely and produce various cell types, the application range of fat fillers has gradually expanded to the field of regenerative medicine.^23-25^

Facial fat grafting is used for facial rejuvenation to address issues such as sun damage, volume deficiency (eg, orbital, tear trough, and temporal depression), skin relaxation, and wrinkles. In addition, fat grafting can be combined with other surgeries such as facelift, blepharoplasty, and laser resurfacing to enhance the effect of facial rejuvenation through multi-modal techniques.^26^ AFG is particularly suitable for treating depressed scars or scars with volume loss, correcting these defects by utilizing the inherent volume enhancement brought by transferred fat.^27^ Moreover, AFG performs well in addressing fibrosis, a common challenge in scar management. It has proven effective in various situations, including scars causing dysfunction, chronic wounds, and fibrotic tissue after radiotherapy. This intervention directly targets fibrotic tissue, promoting its remodeling and reducing the accumulation of pathological fibrous connective tissue.^28^ Rigotti et al were the first to demonstrate that breast cancer patients who received radiotherapy showed significant improvement in symptoms and appearance after fat grafting.^29^ Other studies have also confirmed the positive effects of AFG in breast reconstruction, improving both function and aesthetics.^30^ Ribuffo et al's study showed that 2 separate AFG treatments on the breast 6 weeks after radiotherapy and 3 months before the completion of implant-based breast reconstruction can significantly reduce the risk of skin ulcers and implant exposure.^31^ Other hand symptoms in patients with systemic sclerosis also improved after fat grafting. Studies have shown that AFG to the hand significantly improved hand circulation, Raynaud's phenomenon, hand tightness, modified Rodnan skin score, and disability.^32,33^

In our clinical application, we also found that AFG can not only increase the volume of the mons pubis, labia majora, and anterior and posterior vaginal walls but also alleviate vaginal dryness and dyspareunia to varying degrees. Among them, 3 patients with mild stress urinary incontinence reported a subjective decrease in the number of urinary leaks postoperatively, but this study did not use urodynamic tests such as urethral pressure measurement or ultrasound to verify this effect, which requires further research for confirmation. The authors propose that injecting fat into the anterior vaginal wall may increase the urethral curvature, exert a supporting effect on the urethra, and thereby enable the urethra to withstand a certain level of pressure. The fullness of the mons pubis and labia majora makes women more confident.^34^ Fat grafting to the anterior and posterior vaginal walls can not only make the vagina tighter but also improve women's sexual experience. A study by Lai et al found that AFG improves sexual function by increasing collagen formation, angiogenesis, and estrogen receptors.^18^

During the surgical operation, the subcutaneous area of the mons pubis and labia majora is adipose tissue, making injection very safe. There is a potential space around the vaginal wall without major blood vessels, provided that the injection level is appropriate. During fat grafting to the posterior vaginal wall, the index finger of the nondominant hand is inserted into the patient's vagina to sense the depth and direction of the blunt end of the injection needle, preventing accidental penetration of the rectum. There are differences in fat survival rates. The fat survival rate in the mons pubis and labia majora is relatively high.^35^ Although the authors have noted similarly high levels of fat graft retention, the short timeline and design of this study does not allow us to specifically comment on fat graft survival.

A major challenge in AFG is the variable survival rate of transplanted adipocytes (fat cells), which typically ranges from 30% to 70%.^36^ Poor survival leads to volume loss, asymmetry, or the need for repeat procedures. PRP addresses these issues by (1) providing growth factors, (2) modulating inflammation, (3) supporting adipocyte viability, (4) promoting tissue regeneration and quality, and (5) stem cell activation.^20,37-40^ While promising, PRP's efficacy in AFG is not universally confirmed. Meta-analyses show conflicting outcomes, with some studies reporting significant improvements in graft survival and others showing marginal or no benefit. This may stem from differences in PRP preparation (eg, platelet concentration, activation method), patient factors, or procedural techniques.^41-44^ The PRP concentration and activation protocols of this study need further validation in future research.

This study has several limitations. Firstly, it is a retrospective study with a relatively small sample size (38 cases), which may restrict the generalizability of the findings. Secondly, the lack of a control group makes it hard to compare the efficacy with other treatment modalities. Thirdly, this study did not set up a monotherapy group with AFG alone, making it impossible to clearly define the independent synergistic effect of PRP; future research should conduct multi-center randomized controlled trials to compare the efficacy differences between AFG combined with PRP and AFG monotherapy, while also increasing the sample size to over 100 cases to enhance the extrapolation of results. Fourth, the “ideal appearance” of the vaginal forchet remains context dependent, and our study's definition has limitations that warrant consideration, cultural and personal differences in aesthetic preferences may further shape perceptions of “ideal”—our cohort (predominantly aged 35-50 years, urban residents) prioritized “natural-looking contour” over “tightening” or “symmetry,” which may differ from preferences in other demographics. Moreover, this study has initiated extended follow-up at 12 and 24 months postoperation. Currently, 15 patients have completed the 12-month follow-up, with no observation of fat absorption exceeding 20%. The specific follow-up duration is not mentioned, so long-term outcomes regarding fat retention, functional improvement stability, and potential late complications cannot be fully evaluated. Additionally, subjective evaluations might have introduced bias as objective quantitative indicators for assessing vaginal relaxation and sexual function were not clearly specified. A phone interview for VLQ and FSFI 6 months after discharge, although performed by a professional nurse, might also serve as a potential source of bias when not performed in an anonymized manner. With the progress of research on adipose stem cells, the application of fat grafting will become more extensive, providing a new direction for disease treatment and improvement of quality of life.

## CONCLUSIONS

AFG combined with PRP treatment may effectively improve the aging of female external genitalia and vagina in the short term, as assessed by the VLQ and FSFI scales. Further studies examining the long-term effects of this combined treatment would help to guide future treatment for patients with genitourinary symptoms of menopause.
